# SENP3 regulates the global protein turnover and the Sp1 level via antagonizing SUMO2/3-targeted ubiquitination and degradation

**DOI:** 10.1007/s13238-015-0216-7

**Published:** 2015-10-28

**Authors:** Ming Wang, Jing Sang, Yanhua Ren, Kejia Liu, Xinyi Liu, Jian Zhang, Haolu Wang, Jian Wang, Amir Orian, Jie Yang, Jing Yi

**Affiliations:** Shanghai Key Laboratory of Tumor Microenvironment and Inflammation, Department of Biochemistry and Molecular Cell Biology, Institutes of Medical Sciences, Shanghai Jiao Tong University School of Medicine, Shanghai, 200025 China; Department of Pathophysiology, Institutes of Medical Sciences, Shanghai Jiao Tong University School of Medicine, Shanghai, 200025 China; Department of Biliary-Pancreatic Surgery, Ren Ji Hospital, Shanghai Jiao Tong University School of Medicine, Shanghai, 200127 China; Faculty of Medicine, Cancer and Vascular Biology Research Center, Technion-Israel Institute of Technology, Haifa, 31096 Israel

**Keywords:** SUMOylation, ubiquitination, SENP3, RNF4, Sp1, gastric cancer

## Abstract

SUMOylation is recently found to function as a targeting signal for the degradation of substrates through the ubiquitin-proteasome system. RNF4 is the most studied human SUMO-targeted ubiquitin E3 ligase. However, the relationship between SUMO proteases, SENPs, and RNF4 remains obscure. There are limited examples of the SENP regulation of SUMO2/3-targeted proteolysis mediated by RNF4. The present study investigated the role of SENP3 in the global protein turnover related to SUMO2/3-targeted ubiquitination and focused in particular on the SENP3 regulation of the stability of Sp1. Our data demonstrated that SENP3 impaired the global ubiquitination profile and promoted the accumulation of many proteins. Sp1, a cancer-associated transcription factor, was among these proteins. SENP3 increased the level of Sp1 protein via antagonizing the SUMO2/3-targeted ubiquitination and the consequent proteasome-dependent degradation that was mediated by RNF4. De-conjugation of SUMO2/3 by SENP3 attenuated the interaction of Sp1 with RNF4. In gastric cancer cell lines and specimens derived from patients and nude mice, the level of Sp1 was generally increased in parallel to the level of SENP3. These results provided a new explanation for the enrichment of the Sp1 protein in various cancers, and revealed a regulation of SUMO2/3 conjugated proteins whose levels may be tightly controlled by SENP3 and RNF4.

## INTRODUCTION

Ubiquitin and the small ubiquitin-like modifier (SUMO) all belong to a type of small post-translational protein modifiers (Gill, 2004; Herrmann et al., 2007; Yeh et al., 2000). An increasing number of proteins have been identified as the substrates of both modifiers (Hunter and Sun, 2008; Ulrich, 2005). SUMOylation and ubiquitination occur on the same protein and even at the same lysine residue. They may lead to opposite consequences in protein stability because SUMOylation competes with ubiquitination and antagonizes ubiquitin-proteasome-mediated degradation (Denuc and Marfany, 2010; Ulrich, 2005). However, a novel class of ubiquitin ligases termed “SUMO-targeted ubiquitin ligases (STUbL)” has been discovered in recent years, and the related human RNF4 homodimer is among the best described STUbL (Abed et al., 2011; Geoffroy and Hay, 2009; Sriramachandran and Dohmen, 2014). Thus, SUMOylation can target proteins for degradation through the ubiquitin-proteasome system (Schimmel et al., 2008). Ubiquitination modifies the substrates that have been precedently SUMOylated, during which STUbLs add ubiquitin directly at the SUMO chain moieties or other lysine sites of the substrates (Geoffroy and Hay, 2009). This SUMO-targeted proteolysis is important for a variety of cellular processes (Praefcke et al., 2012).

The SUMO family in humans primarily includes SUMO1, SUMO2 and SUMO3 (Bossis and Melchior, 2006; Yeh et al., 2000). SUMO2 and SUMO3 are 96% similar to each other and are thus called SUMO2/3 (Geiss-Friedlander and Melchior, 2007; Yeh et al., 2000). SUMO1 and SUMO2/3 are conjugated to substrates through an enzymatic cascade (Bernier-Villamor et al., 2002; Bossis and Melchior, 2006; Sampson et al., 2001). The conjugation of SUMO to an internal lysine (K) residue of another SUMO can lead to the formation of substrate-attached SUMO chains (poly-SUMOylation) (Bylebyl et al., 2003; Tatham et al., 2001). It has been believed that SUMO2/3 form SUMO chains more effectively than SUMO1 (Matic et al., 2008). SUMOylation can be reversed, i.e., single SUMO moieties or SUMO chains can be de-conjugated, by a family of SUMO proteases or SENPs in mammals (Drag and Salvesen, 2008). The SENPs family consists of SENP1–SENP3 and SENP5–SENP7, where SENP3, -5, -6 and -7 have preferences for SUMO2/3 de-conjugation (Gong and Yeh, 2006) and SENP6 and SENP7 have specificity for SUMO chains (Lima and Reverter, 2008).

Therefore, the STUbLs and SUMO proteases can both affect the dynamics and consequences of SUMOylation (Sun et al., 2007). The connection between these two types of enzymes has been recently demonstrated (Mukhopadhyay et al., 2010; Wang et al., 2006). However, the relationship between the SUMO proteases and the STUbLs remains largely obscure. For example, SUMO proteases are assumed to de-SUMOylate STUbLs’ substrates destined for the proteasome before or after the substrates’ arrival at the proteasome (Hickey et al., 2012); however, the SUMO proteases can de-SUMOylate the substrates at the very beginning of the process, i.e., before they become the substrates of the STUbLs (Mukhopadhyay et al., 2010). The moment when SENPs perform de-SUMOylation, i.e., before or after the conjugation of ubiquitin by STUbLs, needs to be clarified. Although the list of substrates for mammalian STUbL RNF4 has grown rapidly in recent years (Sriramachandran and Dohmen, 2014) and novel STUbL, Arkadia (RNF111), has been discovered (Erker et al., 2013; Poulsen et al., 2013), there are limited examples of the SENP regulation of SUMO-targeted proteolysis mediated by mammalian STUbL (Mukhopadhyay et al., 2010). Most of the STUbLs bear multiple SUMO interaction motifs (SIMs) that mediate cooperative binding to multiple SUMO units, thereby providing a preference for substrates with SUMO chains (Tatham et al., 2008; Uzunova et al., 2007). Thus, the STUbls have a preference of substrates conjugated with SUMO2/3 that form SUMO chains more effectively than with SUMO1 (Matic et al., 2008). RNF4 is shown to act in a SUMO2/3-dependent manner (Sriramachandran and Dohmen, 2014). However, among the SUMO2/3 specific proteases, only SENP6 is reported to stabilize its substrate by antagonizing RNF4 (Mukhopadhyay et al., 2010). It is not clear whether other SUMO2/3 specific SENPs are involved in the STUbL-mediated protein ubiquitination/degradation.

Specificity protein 1 (Sp1) is a ubiquitously expressed basal transcription factor that regulates a large panel of target genes (Wang et al., 2003). Increased Sp1 accumulation and transcriptional activity have been found in various cancerous tissues and cell lines (Honda et al., 2006; Kanai et al., 2006; Lou et al., 2005). Sp1 is regulated by multiple types of post-translational modifications (PTM) (Chang and Hung, 2012). Previous research has suggested that the SUMO1 conjugation of Sp1 facilitates the interaction between Sp1 and the subunit of proteasome rpt6, resulting in RNF4-dependent ubiquitin-proteasomal degradation (Wang et al., 2008). A recent work has showed that SUMO1 and SUMO2/3 can differentially regulate the role of Sp1 (Gong et al., 2014). Thus, we wondered whether Sp1 can be conjugated with SUMO2/3 and be a substrate of SENP3, as we have previously shown that the protein level of SENP3, a SUMO2/3 specific protease, is increased in tumor tissues and under various stress conditions (Han et al., 2010; Huang et al., 2009; Yan et al., 2010).

The present study investigated the role of SENP3 in the global protein turnover related to SUMO2/3-targeted ubiquitination, and focused in particular on the SENP3 regulation of the stability of Sp1. Our findings provide a new explanation for the enrichment of Sp1 protein in cancers and suggest a global regulation of SUMO2/3 conjugated proteins whose levels may be tightly controlled by SENP3 and RNF4.

## RESULTS

### SENP3 impairs the global ubiquitination profile

**Figure 1:**
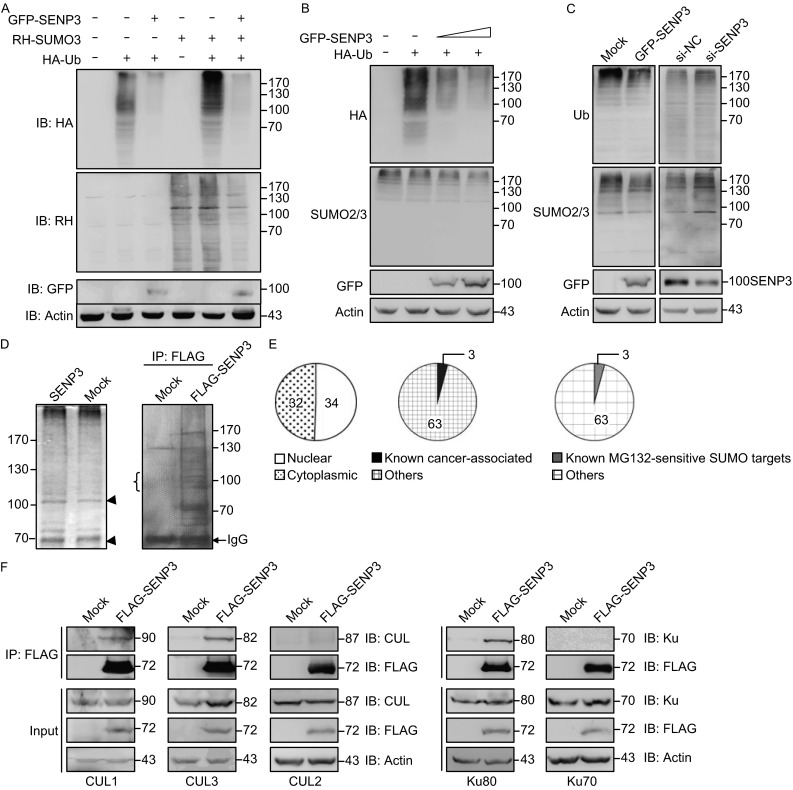
**SENP3 impairs the global ubiquitination profile**. (A) HEK293T (293T) cells were transfected with RH-SUMO3, HA-Ubiquitin (HA-Ub) and GFP-SENP3 for 48 h. The ubiquitin and SUMO3 conjugations of global proteins were determined by immunoblotting (IB) using the antibodies against the tags. (B) 293T cells were transfected with HA-Ubiquitin (HA-Ub) and the increased doses of GFP-SENP3 for 48 h. The ubiquitin and endogenous SUMO2/3 conjugations of global proteins were determined by IB using the antibodies against the tags and SUMO2/3. (C) 293T cells were transfected with GFP-SENP3 or GFP vector, or transfected with SENP3 siRNA or non-specific (NS) siRNA for 48 h. The endogenous ubiquitin and SUMO2/3 conjugations of global proteins were determined by IB using the indicated antibodies. (D) 293T cells were transfected with SENP3 or vector for 48 h. The cell lysates were run through 10% SDS-PAGE and the gel was silver stained. The increased proteins at major band of approximately 100 kDa (arrowhead) were examined by mass spectrometry (MS). 293T cells were transfected with vector FLAG-pcDNA or FLAG-SENP3 and Co-IP was performed using anti-FLAG antibody. The gel was silver stained and the proteins of interest at bands of approximately 100 kDa (braces) were examined by MS. (E) The proteins identified by MS were classified into the nuclear or cytoplasmic proteins and cancer-associated or non-associated proteins by a bioimformatic analysis based on the data banks (Subcellular localization analysis: Gene Ontology (Cellular Component) annotation with DAVID; and Cancer gene exploration: COSMIC database (Version 68) of Sanger), further classified into known or unknown MG132-sensitive SUMO targets based on literatures. (F) 293T cells were transfected with FLAG-pcDNA or FLAG-SENP3 for 48 h, then transfected cells were lysed for Co-IP with M2 beads and detected by Cullin1, Cullin2, Cullin3 (abbreviated as CUL1,2,3), XRCC5 (Ku80) and Ku70 antibodies

We tested whether SENP3, a SUMO2/3-specific protease, could affect global ubiquitination. HEK293T cells were co-transfected with ubiquitin (Ub), SUMO3 and SENP3, respectively or in combination. Immunoblotting of whole cell lysates showed that global ubiquitin conjugations, in particular those at higher molecular weight, were dramatically increased when SUMO3 was overexpressed (Fig. 1A, compare lane 5 with lane 2 in the upper panel). Co-transfecting SENP3 with SUMO3 decreased the global SUMO3 conjugations and concomitantly decreased the global ubiquitin conjugations (Fig. 1A, compare lane 3 with lane 2, and lane 6 with lane 5). Furthermore, the overexpression of an increasing dose of SENP3 in cells that expressed exogenous ubiquitin and endogenous SUMO2/3 led to a marked decrease of ubiquitin conjugations in a SENP3 dose-dependent pattern (Fig. 1B). We then further determined endogenous SUMO2/3 and ubiquitin conjugations in cells with SENP3 overexpression and knockdown. A parallel decrease or increase of SUMO2/3 and ubiquitin conjugations could be observed in these two pairs of cells (Fig. 1C).

The data indicated that the ubiquitination of a population of proteins relies on SUMO3 conjugation, and the de-conjugation of SUMO3 by SENP3 can attenuate ubiquitination. Thus, we wanted to identify proteins that were prevented by SENP3 from undergoing ubiquitin/proteasome-mediated degradation and became stabilized. SENP3 was overexpressed in 293T cells, and the cell lysates were run through electrophoresis. Silver staining showed an enrichment of proteins in the position of 100 kDa and above in SENP3-transfected cells, compared to those in vector-transfected cells (Fig. 1D, left). We took the major band of approximately 100 kDa and examined it with mass spectrometry. Remarkably, the levels of 268 proteins were plausibly increased by SENP3. To search the proteins that were increased through interaction with SENP3, no matter directly or indirectly, we co-immunoprecipitated proteins using the tagged SENP3 and analyzed the bands of approximately 100 kDa by mass spectrometry (Fig. 1D, right). The results showed 360 proteins that had direct or indirect interaction with SENP3. Sixty-six proteins that overlapped in these two groups were then considered to be increased through the possible interaction with SENP3 (Table 1).

**Table 1:** The proteins interacting with SENP3 and probably being increased by SENP3

Nuclear	UBE3A, XAB2, KPNB1, MCM4, NUP93, CDC5L, ELAC2, GTF3C4, NCBP1, TELO2, MAD1L1, MCM3, DHX15, DDX27, CUL1, UBTF, VCP, ZW10, TRIM28, MATR3, NSUN2, CUL3, HNRNPUL1, RRP1B, LAS1L, ZC3H14, XRCC5, MCM5, NCAPH, MCM7, SSRP1, SP1, MRE11A, DDX17
Cytoplasmic	PSMD2, COPG1, COPG2, PYGL, PYGB, GANAB, DRIP4, PDCD6IP, EEF2, HSP90B1, TRA1, MOGS, GCS1, VPS35, AP1G1, UFL1, RRM1, CSDE1, SEC63, QARS, ALDH18A1, MAGED1, PFKP, TFRC, GPHN, HSP90AA1, DKFZp667N107, IMMT, LEPRE1, HSP90AB1, LOD2, TARS
Known cancer-associated	TFRC, GPHN, SP1
MG132-sensitive SUMO targets	NSUN2, MCM7, SP1

SENP3, as a nucleolar protein, preferentially, nevertheless not exclusively, interacts with nuclear proteins (Drag and Salvesen, 2008; Gong and Yeh, 2006; Han et al., 2010; Hickey et al., 2012; Huang et al., 2009; Mukhopadhyay and Dasso, 2007; Yan et al., 2010), and has increased levels in various cancer tissues (Han et al., 2010; Hickey et al., 2012). Therefore, these proteins were examined by a bioinformatic analysis based on the databases (Gene Ontology Cellular Component annotation with DAVID, and Cancer gene exploration COSMIC database Version 68) of Sanger (Forbes et al., 2011; Huang da et al., 2009; Keshava Prasad et al., 2009; Shepherd et al., 2011) and classified into subtypes according to whether they were nuclear proteins and known cancer-associated proteins (Fig. 1E, left and middle). Moreover, we searched for those that had been reported in literature (Gong et al., 2014; Schimmel et al., 2008; Wang et al., 2008) to be stabilized by the proteasome inhibitor MG132 and simultaneously modified by SUMO. Surprisingly, only a few were known to be regulated by SUMO-targeted ubiquitination (Fig. 1E, right). We further performed co-immunoprecipitation (co-IP) assays using available antibodies to corroborate whether the listed nuclear proteins indeed had physical interaction with SENP3. Cullin family members, CUL1 and CUL3, and XRCC5 (Ku80) were proven to probably bind with SENP3, although only CUL3 had a marked increase in cell lystates. CUL2 and Ku70 were tested as the negative controls (Fig. 1F).

These data indicated an indispensible role of SENP3 in regulating the SUMO2/3-targeted ubiquitination/degradation on a global scale, in which the turnover of a number of cancer-related nuclear proteins may be controlled.

### SENP3 regulates the Sp1 protein level through interfering with the SUMO2/3-targeted ubiquitin/proteasome pathway

**Figure 2:**
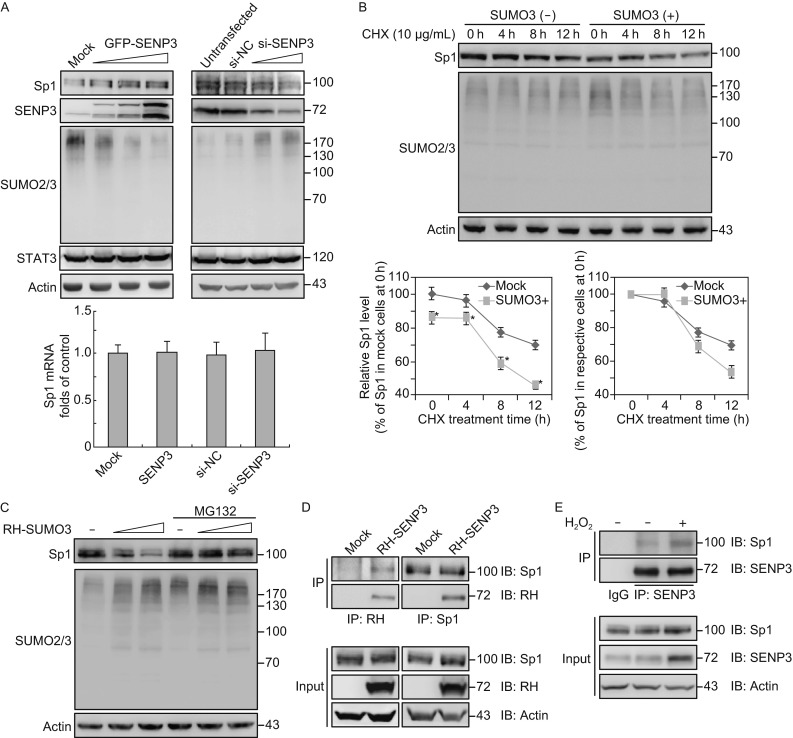
**SENP3 regulates the Sp1 protein level through interfering with the SUMO2/3-targeted ubiquitin/proteasome pathway**. (A) 293T cells were transfected with the increasing doses of GFP-SENP3 for 48 h or the oligonucleotides for SENP3 siRNA for 72 h. Sp1 protein levels were determined by IB using the antibody against Sp1. Global SUMO2/3 conjugation was detected by IB with antibody against SUMO2/3. STAT3, as a negative control, was also detected by IB with its antibody (upper). Sp1 mRNA levels were determined by qRT-PCR at the same time (bottom). (B) 293T cells were transfected with RH-SUMO3 or pcDNA3 and then were exposed to the protein synthesis inhibitor cycloheximide (CHX) for indicated time. Sp1 protein levels were determined by IB using the antibody against Sp1. Global SUMO2/3 conjugation was detected by IB (upper). The Sp1 levels were normalized to the actin levels at each time point, being quantified by intensity analysis with ImageJ software. The relative Sp1 levels were calculated and displayed in two ways. The Sp1 level in the mock-transfected cells at the 0 h of CHX treatment was taken as 100%, and those at each time point in the mock-transfected cells and SUMO3-expressing cells were compared (bottom left). Alternatively, the Sp1 levels in the mock-transfected cells and SUMO3-expressing cells at the zero time point of CHX treatment were taken as 100% respectively, and those at each time point were compared with their own beginning levels, showing the differences when Sp1 level in the SUMO3-expressing cells reached to 50% of its beginning level (right plot). *, *P* < 0.05. (C) 293T cells were transfected with RH-SUMO3 for 48 h in the presence/absence of MG132 (10 μmol/L) for the last 10 h. Sp1 protein levels were determined by IB using the antibody against Sp1. Global SUMO2/3 conjugation was detected by IB. (D) 293T cells were transfected with RH-SENP3 or vector for 48 h. The Sp1 interactions with exogenous SENP3 were determined by immunoprecipitation (IP) using anti-Sp1 and anti-RH antibodies respectively and IB as indicated. (E) Co-IP using anti-Sp1 antibody or IgG were performed in 293T cells with or without H_2_O_2_ exposure, and precipitation of endogenous SENP3 was determined by IB with anti-SENP3 antibody

One of the molecules that well matched to all three aforementioned criteria was Sp1 (Table 1). To confirm the association of SENP3 with Sp1, we determined Sp1 mRNA and protein levels in 293T cells where SENP3 was dose-increasingly overexpressed or knocked-down. Consistently, the protein level of Sp1 was increased by overexpression of SENP3 in a dose-dependent manner. Knocking-down the SENP3 expression by siRNA but not the control siRNA led to a robust decrease of the Sp1 protein (Fig. 2A, upper). The mRNA level of Sp1 was not changed by overexpression or silencing of SENP3 (Fig. 2A, bottom), indicating that the SENP3 promoted the stabilization of Sp1 at post-transcription level, likely PTM level.

To confirm the speculation that SENP3 and SUMO2/3 may have the opposite effect on regulating Sp1 stability, we transfected 293T cells with SUMO3, and added cycloheximide (CHX) to suppress the protein synthesis. Immunoblotting results showed that the existed endogenous Sp1 protein was at a lower level at each time point of CHX treatment in cells overexpressing SUMO3, compared to the cells expressing mock DNA (Fig. 2B, upper and bottom left) and its half-life was apparently shorter in these cells (Fig. 2B, bottom right). In addition, the endogenous Sp1 protein level declined along with the dose of SUMO3, and this decline was attenuated in the presence of proteasome inhibitor MG132 (Fig. 2C).

We next examined whether SENP3 physically interacted with Sp1. Towards this end, 293T cells were transfected with RH-SENP3, and endogenous Sp1 was detectable in the immunoprecipitates. In parallel, when the antibody against Sp1 pulled-down endogenous Sp1, the exogenous SENP3 was detectable in the immunoprecipitates (Fig. 2D). As previously we showed that SENP3 protein accumulates under oxidative stress, the cells were exposed to hydrogen peroxide (H_2_O_2_) to induce an increase of endogenous SENP3. The interaction of both proteins was further confirmed in their endogenous forms by co-immunoprecipitation assay. In addition, an increase of Sp1 protein level and increase of Sp1-SENP3 interaction were observed under the H_2_O_2_ treatment when SENP3 was induced to accumulate to some extent (Fig. 2E).

These results demonstrated that the turnover of Sp1 protein is regulated by SUMO3 through the ubiquitin/proteasome pathway, and SENP3 up-regulates Sp1 protein level likely through a physical interaction.

### SENP3 catalyzes the de-conjugation of SUMO2/3 of Sp1

**Figure 3:**
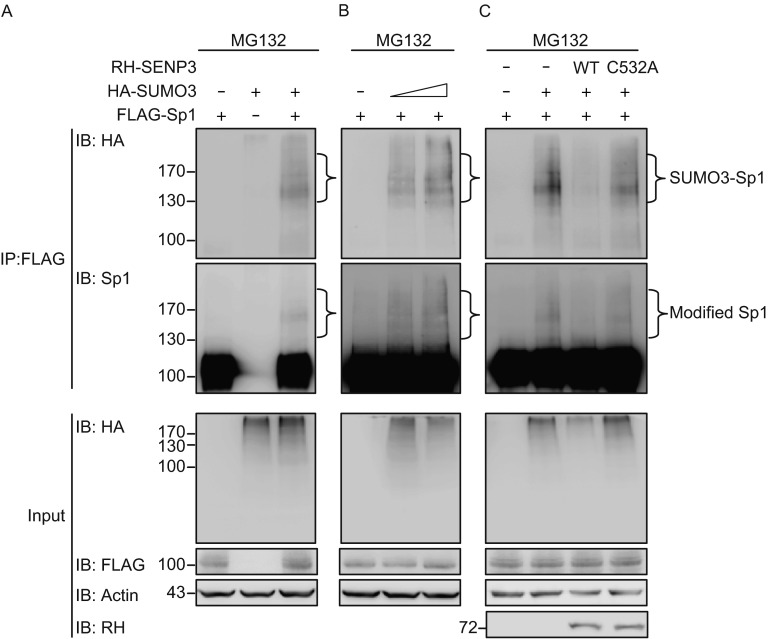
**SENP3 catalyzes the de-conjugation of SUMO2/3 of Sp1**. (A–C) 293T cells were transfected with FLAG-Sp1, HA-SUMO3, with or without SENP3 or mutant for 48 h, and treated with 10 μmol/L MG132 for the last 10 h. The SUMOylation of FLAG-Sp1 was determined by co-IP using M2 beads and IB using the anti-HA and anti-Sp1 antibodies. RH-SUMO3 transfection was with a concentration gradient in (B). RH-SENP3 and RH-SENP3 mutant C532A was co-transfected in (C)

Sp1 was reported to undergo SUMO1 conjugation that was displayed as a single band (Spengler and Brattain, 2006; Wang et al., 2008). Thus we monitored the SUMO2/3 conjugation pattern of Sp1 using a denaturing immunoprecipitation assay in 293T cells co-expressed with FLAG-Sp1 and HA-SUMO3 in the presence of MG132. Exogenous Sp1 pulled-down by FLAG displayed a smear-like SUMO3 conjugation pattern with multiple bands mostly located at molecular weights higher than 130 kDa, confirming that the SUMO3 conjugates of Sp1 were one or multiple polySUMO chain(s) (Fig. 3A). These smear-like multiple bands could be confirmed as the specific SUMO3 conjugates of Sp1 because they were enhanced gradually in a SUMO3 dose-dependent manner (Fig. 3B).

Next, we sought to verify that SUMO3-conjugated Sp1 was the substrate of SENP3. The SUMO3 pulled-down from Sp1 was decreased by SENP3. The SENP3 C532A mutant lacking the de-SUMOylation activity did not lead to the decrease of SUMO3 conjugation (Fig. 3C). These results demonstrated that Sp1 can be modified by SUMO3 and SENP3 is a de-SUMOylating enzyme for Sp1 that removes these conjugates.

### SENP3 can regulate the ubiquitination of Sp1 by abrogating Sp1 interaction with RNF4

**Figure 4:**
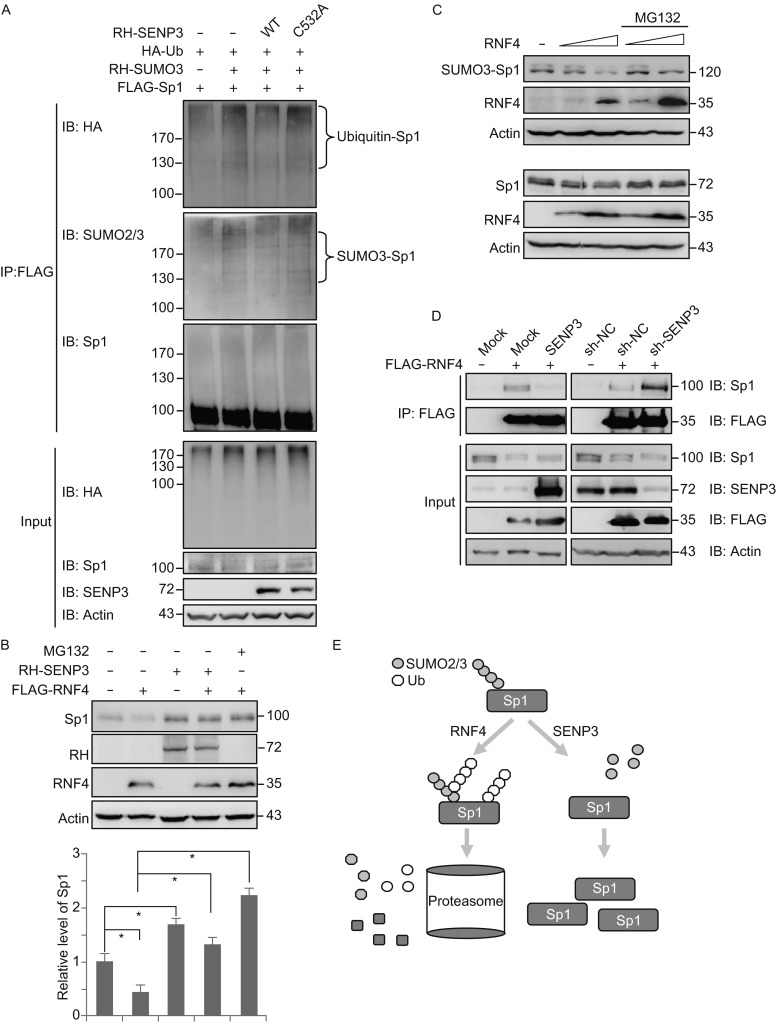
**SENP3 can regulate the ubiquitination of Sp1 by abrogating Sp1 interaction with RNF4**. (A) 293T cells were transfected with HA-ubiquitin (Ub), RH-SUMO3, FLAG-Sp1 and RH-SENP3 or mutant for 48 h, and treated with 10 μmol/L MG132 for the last 10 h. The Ubiquitination and SUMOylation of FLAG-Sp1 were determined by co-IP using M2 beads and IB using the anti-HA, anti-SUMO2/3 and anti-Sp1 antibodies. (B) 293T cells were transfected with RNF4 and RH-SENP3 in the presence/absence of MG132. Sp1 protein levels were determined by IB using the antibody against Sp1. Sp1 levels were quantified by intensity analysis and shown with relative levels in the cells to those in the mock-transfected cells. *, *P* < 0.05. (C) 293T cells were transfected with SUMO3-Sp1 fusion protein with dose-increased RNF in the presence/absence of MG132. The protein levels of overexpressed (upper) Sp1 or endogenous (bottom) were determined by IB using the antibody against Sp1. (D) 293T cells and 293T cells stably expressing non-specific shRNA (sh-NC) or SENP3 shRNA (sh-SENP3) were transfected with SENP3 and FLAG-RNF4 respectively for 48 h. The Sp1 interactions with exogenous RNF4 were determined by co-IP using anti-FLAG antibody and detected by IB using the anti-Sp1 and anti-FLAG antibodies. (E) A model we suggested to illustrate a role of SENP3 in antagonizing RNF4-mediated ubiquitination/degradation of Sp1 and leading to Sp1 stabilization

To explore the association of SUMO2/3 modification and degradation of Sp1, we transfected 293T cells with ubiquitin, SUMO3 and Sp1, respectively or in combination, in the presence of MG132. The denaturing co-IP assays showed that the ubiquitin conjugates on Sp1 were readily detectable when the cells were co-transfected with Ub and SUMO3, but were significantly decreased when the cells were co-transfected with SENP3 in addition to Ub and SUMO3. The SENP3 C532A mutant that lost de-SUMOylating activity did not change the Ub conjugation of Sp1 (Fig. 4A).

We next examined the Sp1 level in the cells that were transfected with SENP3 and a STUbL, RNF4. In the absence of MG132, RNF4 apparently decreased the Sp1 protein level, while SENP3 made Sp1 increase. In the presence of MG132, RNF4 could no longer decrease the protein levels of Sp1 (Fig. 4B). These data indicated that SENP3 and RNF4 played opposing roles in the event of ubiquitin/proteasome-mediated degradation of Sp1. Because RNF4 has been shown to recognize SUMO1-conjugated Sp1 and mediate SUMO1-targeted ubiquitination of Sp1 (Wang et al., 2008), we constructed a SUMO3-Sp1 fusion protein. We found that in cells overexpressing an increasing dose of RNF4, the SUMO3-Sp1 fusion protein was gradually degraded (Fig. 4C, upper), with a pattern similar to that of the endogenous wild-type Sp1 (Fig. 4C, bottom). These data confirmed that RNF4 does recognize the SUMO2/3 conjugates of Sp1 and serve as a SUMO2/3-targeted ubiquitin E3 ligase for Sp1.

Next, we examined how SENP3 could antagonize the RNF4-mediated Sp1 ubiquitination, and whether the SENP3-mediated de-conjugation of SUMO2/3 from Sp1 led to the disruption of the Sp1-RNF4 interaction. Co-IP was conducted in cells that were transfected with FLAG-RNF4 and with SENP3-overexpressing or knocking-down plasmids. The Sp1-RNF4 binding was dramatically abrogated by SENP3 overexpression, and in contrast, was facilitated in the absence of SENP3 (Fig. 4D). The data verified that SENP3 abrogates the interaction between RNF4 and Sp1, which is apparently a consequence of the de-conjugation of SUMO2/3 from Sp1. Taken together we suggest a mechanism where SENP3 antagonized RNF4-mediated Sp1 ubiquitination and degradation, leading to upregulating of Sp1 protein level (Fig. 4E).

### The level of Sp1 displays an increase in parallel with the level of SENP3 in gastric cancer cell lines and specimens

**Figure 5:**
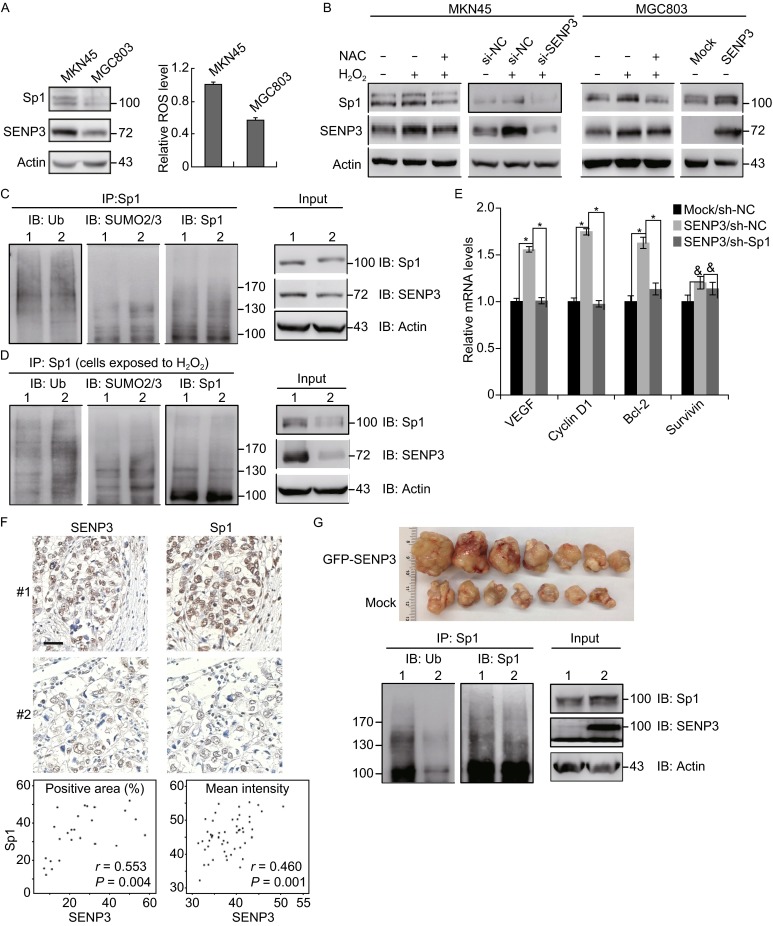
**The level of Sp1 displays an increase in parallel with the level of SENP3 in gastric cancer cell lines and specimens**. (A) The protein levels of SENP3 and Sp1 were determined by IB and ROS levels were detected by flow cytometry using the fluorogenic probe DCFH-DA in gastric cancer cell lines MKN45 and MGC803. (B) MKN45 cells were transfected with non-specific siRNA or SENP3 siRNA for 72 h. MGC803 cells were transfected with RH-SENP3 for 48 h. The cells were pre-treated with 5 mmol/L NAC for 4 h and 200 μmol/L H_2_O_2_ for an additional 0.5 h. SENP3 and Sp1 protein levels were determined by IB. (C) The ubiquitin and SUMO2/3 conjugations for endogenous Sp1 were determined by co-IP in MKN45 and MGC803 cells in the presence of 10 μmol/L MG132. 1 = MKN45 cells; 2 = MGC803 cells. (D) MKN45 cells stably expressing non-specific shRNA (sh-NC) or SENP3 shRNA (sh-SENP3) were exposed to 200 μmol/L H_2_O_2_ in the presence of 10 μmol/L MG132. The SUMO2/3 and ubiquitin conjugations for endogenous Sp1 were examined by co-IP. 1 = sh-NC; 2 = sh-SENP3. (E) MGC803 cells stably expressing non-specific shRNA (sh-NC) or Sp1 shRNA (sh-Sp1) were transiently transfected with GFP-SENP3 or mock for 48 h. The levels of mRNA of Sp1 target genes, *VEGF*, *Cyclin D1*, *Bcl-2* and *Survivin*, were monitored by qRT-PCR. The data represented with mean ± SD from two assays and three replicates in every assay. *, *P* < 0.05. (F) Immunohistochemistry for SENP3 and Sp1 was performed in serial sections derived from different gastric cancer specimens (#1, #2 = two random specimens). The brown staining represents a positive signal. Bar = 30 µm. Image analysis was performed in the paired sections and the percentages of positive areas of both proteins in each specimen were displayed (specimens *n* = 25) (bottom left). The intensities of both proteins in the same areas within one specimen were displayed (field *n* = 50) (bottom right). (G) Human gastric cancer cell line SGC7901 cells stably overexpressing SENP3 or vector were inoculated into nude mice to form tumor xenografts, and the tumors were then recovered, minced into small pieces and implant beneath the serosa of the stomach of other mice to grow for eight weeks (upper). All lysates of the tumors in the same group were mixed. The protein levels and ubiquitin conjugations of endogenous Sp1 were evaluated by co-IP and IB using the indicated antibodies. 1 = mock DNA-expressing tumor lysates; 2 = SENP3-overexpressing tumor lysates (bottom)

Both SENP3 and Sp1 may over-accumulate in certain types of human cancers (Honda et al., 2006; Kanai et al., 2006; Lou et al., 2005). Therefore, we tested if the parallel association between Sp1 and SENP3 levels in an *in vitro* cell system reflects the situation in cancer cells and patient-derived tissues. Screening several human gastric cancer cell lines, we found that the MKN45 and MGC803 lines exhibit marked differences in their SENP3 levels, and also had differences in their Sp1 levels (Fig. 5A, left). Our previous study demonstrated that the SENP3 protein was stabilized by reactive oxygen species (ROS) (Huang et al., 2009); therefore, we measured the ROS level in this pair of cell lines. Compared to MGC803, MKN45 had higher ROS levels and expressed more SENP3 and Sp1 proteins (Fig. 5A, right). The exposure of cells to hydrogen peroxide (H_2_O_2_) for 0.5 h resulted in an increase of the endogenous SENP3 protein level and a simultaneous increase of the endogenous Sp1 protein level. The antioxidant NAC could reverse the ROS-induced SENP3 increase and simultaneously block the Sp1 increase. These effects of ROS-dependent SENP3 regulation remained true in both cell types (Fig. 5B). Moreover, to directly test the role of SENP3 in these lines, SENP3 level was interfered by knockdown in MKN45 that had a higher basal SENP3 level, and by overexpression in MGC803 that had a lower basal SENP3 level. The results showed that when SENP3 was overexpressed, the Sp1 level was increased; in contrast, when SENP3 was knocked-down by siRNA, the ROS-induced Sp1 increase was abolished (Fig. 5B). These results strongly indicated a redox-dependent regulatory role of SENP3 in Sp1 protein level in gastric cancer cells.

Furthermore, the SUMO2/3 and ubiquitin conjugations for endogenous Sp1 were examined in MKN45 and MGC803 cells. Co-IP assays showed that MKN45 had lower levels of the SUMO2/3 and ubiquitin conjugates for Sp1 than MGC803, which linked to a higher SENP3 level and might explain a higher Sp1 level in MKN45 cells (Fig. 5C). MKN45 cells with the intact or silenced SENP3 were then exposed to H_2_O_2_ and MG132 treatments. The results of co-IP indicated that SENP3 mediated H_2_O_2_-induced decreases of SUMO2/3 modification and ubiquitination of Sp1 in the endogenous condition (Fig. 5D).

To determine if the transcription activity of Sp1 was affected by SENP3, we measured mRNA levels of several target genes of Sp1. VEGF, cyclin D and Bcl-2 were obviously transcriptionally upregulated in SENP3 overexpression cells, but these upregulations were almost completely prevented in cells with SENP3 overexpression plus Sp1 knockdown. The transcription of survivin was slightly affected (Fig. 5E).

We next explored whether the expression levels of SENP3 correlated with the expression of Sp1 in human gastric carcinoma specimens using immunohistochemistry performed on the continuous sections of 21 surgically dissected tissues derived from gastric cancer patients. The positive immunohistochemical stain for Sp1 in the nuclei was visible in the majority of cancerous epithelial cells. In parallel, SENP3 stain was markedly positive in the same samples and the same areas. Some tissues displayed a weaker Sp1 stain, whereas the SENP3 stain was also weaker in the same areas (Fig. 5F, upper). The analysis of these paired sections demonstrated a linear correlation between the positive areas of both proteins in each specimen (Fig. 5F, bottom left). In addition, the positive intensity of both proteins in the same areas within one specimen displayed a linear correlation (Fig. 5F, bottom right).

Finally, the correlation between SENP3 and Sp1 levels was evaluated in the xenografts of human gastric cancers grown in the nude mice. SGC7901 gastric cancer cells that had moderate SENP3 level were stably transfected with SENP3. The tumors in the stomachs in the SENP3 overexpression group were larger than those in the control (Fig. 5G, upper). All lysates of the tumors were mixed in the same group to compare the protein levels and ubiquitin conjugates of Sp1 between two groups. IB showed that endogenous Sp1 levels were higher in SENP3 overexpressed tumors. Accordingly, the co-IP showed that endogenous ubiquitin conjugates for Sp1 were less prominent in SENP3 overexpressing tumors (Fig. 5G, bottom). These results provided evidence that SENP3 promotes the growth of gastric cancers, in which Sp1 protein level is increased by SENP3.

## DISCUSSION

A quantitative proteomic study revealed a preference for SUMO2-conjugated proteins being subsequently ubiquitinated and degraded by the proteasome, in which ubiquitin was found to accumulate in purified SUMO2 conjugates but not in SUMO1 conjugates. In addition, endogenous SUMO2/3 conjugates, but not endogenous SUMO1 conjugates, were found to accumulate in response to proteasome inhibitors (Schimmel et al., 2008). The first *in vivo* substrate identified for the SUMO-targeted ubiquitination pathway was mammalian PML, where SUMO2/3-targeted ubiquitination catalyzed by RNF4 was required for PML degradation (Lallemand-Breitenbach et al., 2008). RNF4 was shown to act on substrates in a SUMO2/3-dependent manner (Sriramachandran and Dohmen, 2014). However, only SENP6 and no other SUMO2/3 specific SENPs has been reported to stabilize their substrates (Mukhopadhyay et al., 2010). Our present study demonstrated that SENP3 played a crucial regulatory role in RNF4-mediated proteolysis that depended on the SUMO2/3-targeted ubiquitination. Surprisingly, this regulation was seen on a global scale; hundreds of proteins might be governed under this regulation. Our previous study identified SENP3 as a redox sensitive molecule that increased its abundance under various conditions of oxidative stress (Huang et al., 2009). Therefore, a profound change in global protein turnover could be predicted following the increase of SENP3 under oxidative stress-related physiological and pathological scenarios including cancers.

Currently, it is unclear how SENPs acts on RNF4’s substrates destined for the proteasome. When a SENP de-SUMOylates these substrates, it has been assumed to do so before or after the substrates’ arrival at the proteasome (Hickey et al., 2012). In these cases, SENP might cooperate with RNF4 to achieve the elimination of the SUMOylated proteins. Alternatively, if de-SUMOylation occurred before the addition of ubiquitin by RNF4, the SENP would antagonize RNF4, protecting the substrates from destruction. Recent work by Mukhopadhyay et al. has demonstrated that SENP6 stabilizes kinetochore component CENP-1 by specifically shortening SUMO2/3 chains on CENP-1 and antagonizing RNF4 (Mukhopadhyay et al., 2010). In the present study, we show that SENP3 could remove all polySUMO2/3 conjugates from Sp1 and from many other substrates, and simultaneously remove ubiquitin conjugates. Our data suggest that SENP3 can antagonize RNF4’s action through a similar way that used by SENP6, but most likely acts at a basic step, i.e., removes polySUMO chains at the SUMO2/3 conjugate “roots”. In addition, we clearly demonstrate that the SENP3-catalyzed de-conjugation of SUMO2/3 from Sp1 prevents RNF4 from recognizing and binding to Sp1. This confirms the role of SENPs as antagonistic regulators for the substrates of RNF4 and provides the biochemical evidence for a direct action of both SENP and RNF4 with their common substrates.

The transcription factor Sp1 is important for the expression of thousands of housekeeping genes (Chang and Hung, 2012). Although Sp1 has been considered constitutively activated in basal gene expression regulation, a variety of PTMs and protein-protein interaction have been shown to affect its activity and content, especially under specific cellular processes (Wang et al., 2011).

Sp1 is subjected to several PTMs, which modulates the protein level, the DNA-binding activity or the transactivation potential of Sp1 (Chang and Hung, 2012). The PTMs reported to date mainly include phosphorylation (Hung et al., 2006; Tan and Khachigian, 2009), acetylation (Hung et al., 2006; Waby et al., 2010), glycosylation (Han and Kudlow, 1997; Ozcan et al., 2010; Vij and Zeitlin, 2006), ubiquitination (Wang et al., 2011), SUMOylation (Su et al., 1999; Wang et al., 2008; Wang et al., 2011), ribosylation (Bouwman and Philipsen, 2002) and oxidation (Sang et al., 2011). Confusingly, evidence and conclusions are diverse and contradictory for the PTM modulation of the Sp1 protein level. Kudlow and colleagues provided early evidence that imply that O-linked glycosylation protects Sp1 from proteolysis (Han and Kudlow, 1997; Wells et al., 2001). In accordance with these *in vitro* results, Spengler and Brattain demonstrated *in vivo* data that a cAMP activator facilitates the production of cleavage fragments. Their study identified Sp1 as a substrate for SUMO1 modification and showed an inverse relationship between SUMO1-modified Sp1 and N-terminally cleaved Sp1 (Spengler and Brattain, 2006). Subsequently, they showed that phosphorylation mediates Sp1 de-SUMOylation and destabilization during mitosis (Spengler et al., 2008). Differently, Hung and colleagues showed that phosphorylation shields Sp1 from the ubiquitin-dependent degradation during mitosis (Chuang et al., 2008). This group also found that modification of Sp1 by SUMO1 at lysine 16 facilitates Sp1 degradation, and SUMOylation of Sp1 is attenuated during tumorigenesis to increase Sp1 stability (Wang et al., 2008). In addition, their subsequent studies demonstrated that SUMO1 modification of Sp1 could recruit RNF4 as an ubiquitin E3 ligase that subjects SUMOylated Sp1 to proteasomal degradation (Wang et al., 2011). A recent study by Gong and colleagues demonstrated that SUMO1 and SUMO2 exert opposing effects on Sp1 transcriptional activity in the mouse lens system (Gong et al., 2014). Therefore, there is no consistency in the relationship between the SUMO modification and protein stability of Sp1.

In the present study, we used approaches of co-overexpressing and silencing SUMO3, ubiquitin and/or SENP3 to verify the relationship between SUMO modification and the protein stability of Sp1. The results clearly demonstrated that SUMO2/3 conjugations facilitated Sp1 degradation through the formation of SUMO2/3-targeted ubiquitination, whereas SENP3, a SUMO2/3-specific protease, stabilized Sp1 via blockage of SUMO-targeted ubiquitination and proteasome-mediated degradation. The importance of the role of SENP3 in Sp1 regulation can be highlighted in two aspects. First, STUbL RNF4 has preference for substrates conjugated with SUMO2/3 that form SUMO chains more effectively than SUMO1 (Matic et al., 2008). Therefore, it is critical to know whether the SUMO2/3 specific SENP(s) naturally play an antagonistic role for RNF4. Our data have shown that SENP3 regulates the Sp1 protein level through the removal of the polySUMO2/3 chains, thus blocking the recognition and binding by RNF4. Second, the protein level of Sp1 must be controlled by a plethora of regulatory mechanisms and may fluctuate along with the physiological or pathological conditions. Which SENP(s) would prevail in the regulation of Sp1 under a given condition depends on the level and enzymatic activity of the SENPs under that condition. Our previous studies have demonstrated that the SENP3 rapidly (within 1 h) increases its own level through a redox-sensitive stabilization under oxidative stress conditions (Huang et al., 2009; Yan et al., 2010) and remains accumulated in many types of human cancer tissues (Han et al., 2010; Yan et al., 2010). Comparatively, no other SENPs are known to be consistently upregulated under stress or cancer-prone conditions. In the present study, we have shown that H_2_O_2_ treatment leads to an increase of Sp1 in the gastric cancer cell line, which is dependent on an induction of SENP3. Therefore, the SENP3 regulation of the Sp1 level may explain that Sp1 is increased under stress conditions and in cancerous tissues.

The overexpression of Sp1 is considered a prognostic indicator for the poor survival of gastric cancer patients (Wang et al., 2003; Yao et al., 2004). A study based on 396 gastric tissue samples confirmed that the expression of Sp1 increases with tumor progression (Lee et al., 2013). A number of microRNA molecules were recently suggested to regulate Sp1 at a post-transcription level (Guo et al., 2013; Liu et al., 2013). However, the mechanisms underlying Sp1 overexpression have not been adequately investigated. We herein show a close correlation between the protein levels of SENP3 and Sp1 in the patient’s gastric specimens, as well as in the nude mouse xenografts of human gastric cancers. Together with the biochemical and molecular biological data generated in cultured cells, these results have provided a novel regulatory mechanism to explain the overexpression of Sp1 in gastric cancers at the post-translation level.

## MATERIALS AND METHODS

### Cell culture and treatments

Gastric carcinoma cell lines SGC-7901, MKN45 and MGC803 cells were cultured in RPMI 1640 medium (HyClone). The HEK293T (293T) cells were cultured in Dulbecco’s modified Eagle’s medium (HyClone). All media were supplemented with 10% newborn calf serum. The cells were maintained at 37°C in a humidified atmosphere with 5% CO_2_. The stable cell lines with SENP3 over-expression were used in the previous work (Ren et al., 2014). To establish the stable MGC803 cell lines bearing Sp1 shRNA (sh-Sp1) or non-specific shRNA (sh-NC), ZsGreen1 co-expressing lentiviral expression vectors were transiently transfected into 293FT cells. After 72 h, the supernatants were harvested to infect MGC803 cells with a final concentration of 10 μg/mL polybrene. Lastly, the ZsGreen1-positive cells were sorted on a FACSAria II flow cytometer (BD Biosciences). The nucleotide sequence in shRNAs against Sp1 was: CCGGCCCAAGTTTATTTCTCTCTTACTCGAGTAAGAGAGAA ATAAACTTGGGTTTTT. The MGC803 cells stably expressing sh-NC or sh-Sp1 were transiently transfected with GFP-SENP3 or mock for 48 h to reach the effects of SENP3 overexpression with Sp1 knockdown simultaneously.

MG132 (Merck) was used to block proteasome activity. When needed, the anti-oxidant N-acetyl-L-cysteine (NAC) (Sigma-Aldrich) was pre-administered for 4 h before other treatments.

### Immunoblotting (IB)

Cells or tissues were lysed. The proteins were separated on 8% or 10% SDS-PAGE gels, and then were transferred to nitrocellulose membranes before determined by the antibodies. The methods were as previously used (Yan et al., 2010).

### Co-immunoprecipitation (co-IP)

Denaturing co-IP was performed to detect the SUMO or ubiquitin conjugations of Sp1. Cells were lysed in 150 μL of the denaturing buffer (50 mmol/L Tris (pH 7.4), 1% SDS) for 30 min. After boiling for 10 min, cell lysates were centrifuged for 15 min at room temperature. Supernatants were mixed with IP buffer (50 mmol/L Tris (pH 7.4), 150 mmol/L NaCl, 1 mmol/L EDTA, 1% Triton X-100) and incubated with specific antibodies as indicated. The proteins were separated from the beads using IB loading buffer, then the supernatants were collected for IB. The routine co-IP was carried out using methods described in our previous studies (Huang et al., 2009).

### Antibodies

The following antibodies were used in this study: mouse monoclonal antibodies against β-actin (Sigma); Rabbit polyclonal antibodies against Sp1 (Santa Cruz Biotechnology); mouse monoclonal [Ubi-1] antibodies against Ubiquitin (Abcam), the rabbit polyclonal antibodies against HA (Abcam), the mouse monoclonal antibodies against GFP (Abcam), mouse anti-RH (Qiagen), the mouse monoclonal antibodies against FLAG (sigma), the rabbit monoclonal antibodies against SUMO2/3 (Cell Signaling Technology), normal rabbit IgG (CST), the rabbit polyclonal antibodies against RNF4 (Abgent), and the rabbit polyclonal antibodies against SENP3 (Protein Techgroup).

### Mass spectrometry

After gel pieces were destained and washed, the spots were incubated in 0.2 mol/L NH_4_HCO_3_ for 20 min and then lyophilized. The in-gel proteins were reduced with dithiothreitol (10 mmol/L DTT/ 100 mmol/L NH_4_HCO_3_) for 30 min at 56°C, then alkylated with iodoacetamide (50 mmol/L IAA/100 mmol/L NH_4_HCO_3_) in the dark at room temperature for 30 min before briefly rinsed and digested overnight in 12.5 ng/mL trypsin in 25mmol/L NH_4_HCO_3_. The peptides were extracted three times with 60% ACN/0.1% TFA. The extracts were pooled and dried completely by a vacuum centrifuge. Experiments were performed on a Q Exactive mass spectrometer that was coupled to Easy nLC (Thermo Fisher Scientific). Data were acquired using a data-dependent top10 method dynamically choosing the most abundant precursor ions from the survey scan (300–1800 m/z) for HCD fragmentation. Survey scans were acquired at a resolution of 70,000 at m/z 200 and resolution for HCD spectra was set to 17,500 at m/z 200. MS/MS spectra were searched using MASCOT engine (Matrix Science, London, UK; version 2.2). Each sample was repeated twice.

### Plasmids and siRNA

The plasmid FLAG-Sp1 was constructed by inserting an Sp1 fragment amplified from pcDNA-Sp1 into *Not*I-*Xba*lI sites in the pcDNA-FLAG vector. The PCR-amplified SUMO3 fragment with *Eco*RI and *Not*I restriction sites from HA-SUMO3 was ligated into FLAG-Sp1 to obtain the FLAG-SUMO3-Sp1 fusion protein. The plasmid FLAG-RNF4 was made by PCR using specific primers from plasmid RNF4 and by subcloning the PCR products into *Bam*HI and *Not*I restriction sites of the pcDNA-FLAG vector. The pEGFP-C1-SENP3 and its mutant constructs have been previously described (Yan et al., 2010).

The siRNA specific for SENP3 and non-specific control siRNA oligonucleotides were synthesized and used as previously (Han et al., 2010).

### Reactive oxygen species (ROS) detection

DCFH-DA (2-dichlorodihydrofluorescein diacetate; Sigma) was used as a ROS-capturing reagent according to the previous method (Cai et al., 2008).

### Real-time quantitative PCR (RT-qPCR)

The total RNA was isolated following the guide of the TRIzol reagent kit (Invitrogen, Carlsbad, CA). The cDNA synthesis was performed on 2 μg of the total RNA with a reverse transcription kit (Promega, Madison, WI). Real-time PCR was conducted on the ABI Prism 7500 system using SYBR Green (Roche Applied Science, Mannheim, Germany) and following the manufacturer’s instructions.

The primers used were listed below.

**Table:** 

**Name of primer**	**Sequence**
Sp1-f	gagaaaacagcccagatgc
Sp1-r	cccttccttcactgtcttt
VEGF-f	agggcagaatcatcacgaag
VEGF-r	gtctcgattggatggcagtag
CyclinD1-f	cgtggcctctaagatgaagg
CyclinD1-r	tgcggatgatctgtttgttc
Bcl-2-f	acaacatcgccctgtggatgac
Bcl-2-r	atagctgattcgacgttttgcc
Survivin-f	ggaccaccgcatctctacat
Survivin-r	gttcctctatggggtcgtca

### Mouse model for orthotopic gastric cancer development

The SGC7901 cells of 1 × 10^7^ were injected subcutaneously into the 6-wk-old Balb/c nu-nu mice. Two weeks later, tumors were harvested and minced into small pieces (1 mm^3^) in RPMI-1640 basal medium. Each tumor piece was placed into a small tissue pocket formed in the middle wall of the greater curvature of the stomach beneath the serosa in another nude mouse, and fixed using a purse string suture with 7-0 absorbable sutures. After tumor implantation, the stomach was relocated into the abdominal cavity followed by abdominal closure (Li et al., 2011) and tumors were allowed to grow for 8 weeks. All procedures were performed under anesthesia with ketamine (50 mg/kg)/midazolam (10 mg/kg) following an approved protocol in conformity with institutional guidelines for the care and use of laboratory animals in Shanghai Jiao Tong University School of Medicine.

### Immunohistochemistry

The paraformaldehyde-fixed and paraffin-embedded sections of human gastric cancer specimens were archived pathologic specimens from the Ren Ji Hospitals and obtained following an institute-approved protocol. The immunohistochemistry for the SENP3 and Sp1 was conducted on the continuous sections using a previous method (Cai et al., 2008). The ratio of the positive area for SENP3 and Sp1 was quantified by the Zeiss KS400 software. The mean optical density of 50 fields of the same size from 3 pairs of pathologic specimens expressing SENP3 and Sp1 stained by immunohistochemistry was detected.

### Statistical analysis

Results presented were derived from at least three independent experiments. All statistical analyses were performed by using the SPSS software package. The relationship between the expression of SENP3 and Sp1 were examined by the Spearman rank correlation coefficient. A *P*-value of <0.05 was considered statistically significant.

## ABBREVIATIONS

co-IP: co-immunoprecipitation
HA: hemagglutinin
IB: immunoblot
NAC: N-acetyl-L-cysteine
NS: nonspecific
PML: promyelocytic leukemia
PTM: post-translational modification
RH: RGS-His
ROS: reactive oxygen species
SENP: sentrin/SUMO proteases
siRNA: small interfering RNA
Sp1: specificity protein 1
STUbL: SUMO-targeted ubiquitin ligases
SUMO: small ubiquitin-like modifier
